# Design, assembly, and tuning of a multipurpose FPV drone: A flexible and low-cost alternative

**DOI:** 10.1016/j.ohx.2025.e00672

**Published:** 2025-07-22

**Authors:** Franco Alessandro Arenas Mamani, Gustavo Bryam Capira Malcoaccha, Marco Antonio Blanco Quicaño, Leonardo Gabriel Prado Gutierrez, German Alberto Echaiz Espinoza, Erasmo Sulla Espinoza, Andres Ortiz Salazar

**Affiliations:** a School of Electronics Engineering, Universidad Nacional de San Agustin de Arequipa, Peru; b Academic Department of Electronic Engineering, Universidad Nacional de San Agustin de Arequipa, Peru; c Department of Computer Engineering and Automation, Universidade Federal do Rio Grande do Norte, Brazil

**Keywords:** FPV drone, Assembly, Betaflight, UAV, Quadcopter, PID tuning

## Abstract

This article details the design, assembly, and tuning of a multipurpose FPV (First Person View) drone designed as a budget-friendly and customizable alternative to professional-grade drone model. Built with a focus on performance, adaptability, durability, and ease of repair, the *APdrone* (All Purpose Drone) incorporates high-quality components such as a carbon fiber frame, brushless motors, and advanced flight controllers. To ensure optimal flight characteristics, a comprehensive tuning process was performed using empirical adjustments, supported by controlled testing environments, to optimize PID parameters ($K_{p}$, $K_{i}$, $K_{d}$) to achieve stable and precise flight performance. Testing demonstrated a flight time of approximately 8 min under standard conditions with a maximum payload capacity of 0.98 kg. Importantly, the *APdrone* offers operational flexibility and significant cost savings (USD 647.09), compared to high-end drones like the DJI Mavic 3 Pro, while maintaining comparable functionality and allowing for customization. To encourage accessibility, reproducibility and further development, open-source design files, including CAD schematics, firmware configurations, and assembly instructions, are provided. The *APdrone* serves as a scalable platform for research, recreational use, and advancements within the UAV (Unmanned Aerial Vehicle) field.

## Graphical abstract

**Figure:**
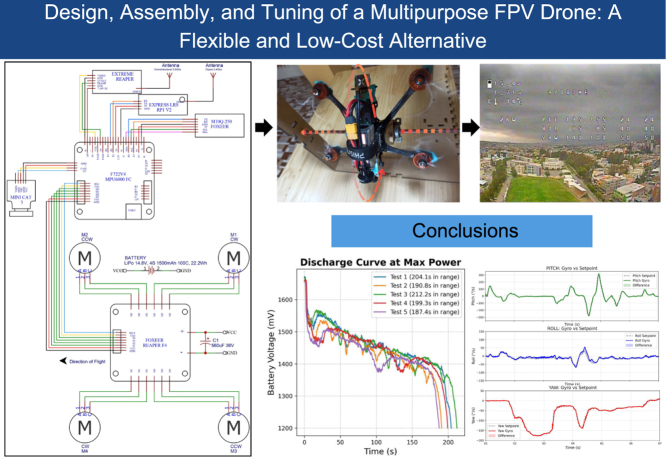

## Specifications table

**Table:** 

Hardware name	*All purpose drone FPV (APdrone)*
Subject area	*Engineering and material science*
Hardware type	*Electrical engineering and computer science*
Closest commercial analog	*Nazgul Evoque F6 V2 O3 GPS*
Open source license	*CC BY 4.0*
Cost of hardware	*$ 647.09 (with all the necessary extra equipment)*
Source file repository	https://doi.org/10.17632/zgsvdtxnfh.2

## 1 Hardware in context

Recent years have witnessed a surge in the use of unmanned aerial vehicles (UAVs) across diverse sectors, driven by their versatility and operational efficiency [1]. However, high-end commercial drones, exemplified by the DJI Mavic 3 Pro, while technologically advanced, present significant drawbacks. Their substantial cost ($2970 USD) [2] and reliance on proprietary software severely limit customization options, effectively restricting their use to professional applications.

This creates a gap for users seeking more accessible and customizable drones without compromising on performance or functionality for professional or semi-professional needs. The FPV (First-Person View) drone community has emerged as a viable alternative, offering customizable configurations that combine flexibility with lower costs. For instance, the “Nazgul Evoque F6 V2 O3 GPS” ($759 USD) [3] provides a significantly more affordable option. However, these alternatives often require the user to purchase essential components like the remote control, batteries, and charging systems separately, increasing both the overall cost and the complexity of setup. This contrasts sharply with the DJI Mavic 3 Pro, where a complete package with all accessories can easily exceed $4890 [4].

Furthermore, operating FPV drones demands a high level of technical expertise. Users must manually adjust critical flight control parameters $K_{p}$, $K_{i}$, and $K_{d}$, to optimize performance based on the specific usage conditions in sectors like environmental monitoring [5] or surveillance [6], etc. This process can be daunting for users with limited technical backgrounds and experience in the field.

This paper is organized as follows. The Hardware Description section outlines the components integrated into the APdrone platform. The Design Files Summary compiles all source code, configurations, and relevant resources. Section 5 lists the materials used, along with their respective sources. The Build Instructions section discusses the availability of components, battery selection, and estimated flight autonomy, followed by step-by-step instructions for assembly, electronic soldering, and frame construction. It also provides guidance on software setup, sensor calibration, and radio control configuration. Subsequently, the methodology for PID parameter tuning and flight data analysis using a dedicated test platform is presented. The final section validates the proposed approach through field experiments, evaluating the drone’s real-world performance.

The contribution of this paper addresses these limitations by introducing the APdrone, a low-cost, high-performance drone designed to compete with existing commercial offerings. The APdrone emphasizes multi-use applications, prioritizing customizability and ease of repair. By incorporating open-source components, it eliminates reliance on expensive proprietary parts and software, making it accessible to a wider user base. We contribute an efficient methodology for determining the optimal values for $K_{p}$, $K_{i}$, and $K_{d}$, enabling precise tuning for any drone.

## 2 Hardware description

The APdrone is a 5-inch custom FPV quadcopter, designed for multipurpose and portable use, with interchangeable propellers. It offers an excellent combination of portability, maneuverability, cost-effectiveness, and versatility. Its compact design and ability to adapt to multiple scenarios make it stand out compared to larger drones, especially when considering budget constraints and the need for mobility.

This drone offers significant advantages compared to high-end commercial drones:

• *Reduced cost:* The APdrone is up to 5 times more affordable than a high-performance drone in its most basic configuration offered by well-known brands. Additionally, its repair and parts replacement costs are much lower, making future upgrades easier.
• *High performance and customization:* The APdrone is assembled with open-access components, without import, use, or commercialization restrictions. Furthermore, it is fully programmable using open-source software, making it compatible with components from other manufacturers if needed.
• *Global accessibility:* All components were imported through the AliExpress platform, an option accessible to much of the world, known for its competitive prices.
• *Adaptability and durability:* This drone is resistant to hostile environments, and its robust structure allows it to withstand rough handling. The APdrone is perfect for those starting to operate UAVs, as it tolerates drops, crashes, and accidents. Combined with its excellent reparability, it is ideal for recreational purposes or experimental research where the drone may fail mid-flight.

The APdrone offers several advantages for research applications:

• *Aerial Inspections and Development Projects:* The high thrust-to-weight ratio and substantial payload capacity make the platform suitable for aerial inspections and for supporting research and development activities across a wide range of operational scenarios.
• *Algorithm Validation and Autonomous Navigation:* Its open-source architecture allows extensive customization, making it ideal for validating control algorithms and conducting experiments in autonomous navigation.
• *High-Performance Maneuvering Studies:* The enhanced thrust capabilities enable aggressive flight maneuvers, providing a reliable platform for analyzing dynamic behaviors and optimizing flight performance under varying conditions.
• *Multi-Scenario Mission Testing:* With mechanical robustness and upgrade flexibility, the system supports complex mission execution across diverse environments, facilitating research that demands adaptable and resilient aerial platforms.

The APdrone features a 5-inch carbon fiber frame, resistant to significant mechanical stresses [7], the use of carbon fiber tubes has demonstrated adequate structural performance in agricultural drone applications, withstanding compression and bending tests with safety factors greater than 1.8 [8]. To maximize the safety and durability of the propellers, it incorporates the use of Foxeer Donut 5145 Toroidal Props, a more stable and robust alternative to the traditional blade design [9]. It is equipped with two pairs of 2507 1800KV brushless motors, providing a wide range of acceleration and torque. The selected flight controller is the *Foxeer F722 V4* (4–8S LiPo), known for its great precision and for allowing the use of various battery types, both for longer flight times and for larger drones [10]. Electronic Speed Controllers (ESCs) are essential components in brushless motor systems, as they convert direct current into a variable and bidirectional voltage signal. Their selection is primarily based on the maximum current they can supply to the motor [11]. *Foxeer Reaper F4 128K* (ESCs), with a peak of 65 A and compatible with an input voltage of 3–8S LiPo, offering a wide range of operations and power for all types of applications [12]; the typical range for an FPV drone is usually 35–55 A, so the 65 A provided by this model is more than sufficient. The drone uses a 1500 mAh 4S battery (12.8 Wh) with a 100 C discharge rate (*capable of providing 100 times its nominal current instantly*) [13]. However, higher-capacity batteries can be used if required; the selected option was considered ideal for facilitating testing.

The APdrone integrates a *Foxeer Mini Cat 3* camera, with a minimum illumination of 0.00001 Lux [14], enabling outstanding performance in low-light conditions. Additionally, it features a *Foxeer M10Q 250* module that provides Global Positioning System (*GPS*) capabilities [15]; thanks to this component, position monitoring and other advanced functionalities are enabled when needed.

For video transmission, a *Foxeer Extreme Reaper* 2.5 W is installed, guaranteeing a wide operational range and equipped with 72 channels [16], offering a stable signal even in environments with interference. Its 2.5 W output power exceeds the average of most *VTX* models on the market. The remote control used is the *Pocket Radio Controller (M2)*, with *EdgeTX* system and compatible with the CC2500 multiprotocol and *ELRS* 2.4 GHz protocols [17]. This setup provides long-range options and the ability to attach an external module to achieve distances of up to 12 km.

For the pilot’s view, the *Eachine EV800D* headset is used, with a resolution of 800 × 480 pixels [18]. The charging system includes the *Imax B6 V3* LiPo battery charger, a multifunctional smart device compatible with various battery types and with a maximum charging power of 80 W [19].

Regarding the drone’s programming and configuration, the *Betaflight* software has been used, recognized for its ease of implementation and wide compatibility [20]. All design files are open-source and available in the corresponding repositories. The PID values were adjusted using a practical method in a controlled test environment, utilizing a sweeping procedure and analysis in *Blackbox Explorer* [21].

## 3 Design files

All the necessary files for the design, assembly, commissioning, tuning, and data processing, as well as the blackbox analysis, can be found in the following repository https://doi.org/10.17632/zgsvdtxnfh.2. Additionally, a set of MP4 video files is included to provide step-by-step visual guidance for key processes such as hardware assembly, Betaflight configuration, data processing, and maintenance. These videos serve as supplementary instructional material to facilitate replication and understanding.

### 3.1 Design files summary

**Table:** 

Design filename	File type	Open source license	Location of the file
Betaflight configuration for F722	TXT file	*CC BY 4.0.*	https://doi.org/10.17632/zgsvdtxnfh.2
Hardware connection diagram	PDF file	*CC BY 4.0.*	https://doi.org/10.17632/zgsvdtxnfh.2
Test rig blueprint for drone axes	DWG File	*CC BY 4.0.*	https://doi.org/10.17632/zgsvdtxnfh.2
Python program for tuning	PY File	*CC BY 4.0.*	https://doi.org/10.17632/zgsvdtxnfh.2
Datasheet of all components	PDF FileS	*CC BY 4.0.*	https://doi.org/10.17632/zgsvdtxnfh.2
FPV drone assembly and setup	MP4 file	*CC BY 4.0.*	https://doi.org/10.17632/zgsvdtxnfh.2
Betaflight configuration guide	MP4 file	*CC BY 4.0.*	https://doi.org/10.17632/zgsvdtxnfh.2
Charging FPV LiPo battery	MP4 file	*CC BY 4.0.*	https://doi.org/10.17632/zgsvdtxnfh.2
FPV drone motor maintenance	MP4 file	*CC BY 4.0.*	https://doi.org/10.17632/zgsvdtxnfh.2
Recording FPV video from Your Goggles	MP4 File	*CC BY 4.0.*	https://doi.org/10.17632/zgsvdtxnfh.2
Processing FPV flight data Python guide	MP4 File	*CC BY 4.0.*	https://doi.org/10.17632/zgsvdtxnfh.2
Supplementary data	MP4, JPG, CSV, RAR	*CC BY 4.0.*	https://doi.org/10.17632/zgsvdtxnfh.2
Moment of inertia calculations	PDF	*CC BY 4.0.*	https://doi.org/10.17632/zgsvdtxnfh.2

## 4 Bill of materials summary

**Table:** 

Designator	Component	Quantity	Cost (USD)		Source	Type of material
			Unitary	Total		
P01	Mark 5 Frame Kit 5 inch Carbon Fiber Structure FPV	1	35.14	35.14	Aliexpress	Carbon Fiber
P02	Foxeer Donut 5145 Props 2-Pairs	1	3.72	3.72	Aliexpress	Polymer
P03	2507 1800 KV Brushless Motor 2-Pairs	1	30.98	30.98	Aliexpress	Metal
P04	Imax B6 V3 Balance Charger 80 W	1	32.90	32.90	Aliexpress	Semiconductor
P05	Eachine EV800D Headset	1	98.34	98.34	Aliexpress	Semiconductor
P06	Pocket Radio Controller (M2)	1	108.80	108.80	Aliexpress	Semiconductor
P07	Foxeer Pagoda Pro 5.8 GHz 3 dBi Omni RHCP SMA Angle 150 mm	1	9.44	9.44	Aliexpress	Semiconductor
P08	Zeee 4S 1500 mAh 14.8 V 100C Lipo Battery	1	17.82	17.82	Aliexpress	Semiconductor
P09	Foxeer Reaper F4 128K 65A BL32 4in1 ESC	1	82.11	82.11	Aliexpress	Semiconductor
P10	Foxeer F722 V4 Flight Controller MPU6000 8S	1	69.77	69.77	Aliexpress	Semiconductor
P11	Foxeer M10Q 250 GPS 5883 Compass	1	21.70	21.70	Aliexpress	Semiconductor
P12	Foxeer 5.8G Reaper Extreme V2 2.5W 72CH VTx	1	48.68	48.68	Aliexpress	Semiconductor
P13	Foxeer Mini Cat 3	1	41.72	41.72	Aliexpress	Semiconductor
P14	MMCX male plug to SMA female connector 10 cm	1	1.45	1.45	Aliexpress	Semiconductor
P15	Custom MDF cutting 8 mm	1	22	22	Maqsoft	Organic Polymer
P16	Bearing 8 mm	3	2.83	8.49	Aliexpress	Steel
P17	Rustproof Steel Smooth Rod 8 mm OD 500 mm long	1	10.19	10.19	Aliexpress	Steel
P18	Vertical Horizontal Bearing Support Seat With Ball 8 mm	2	1.92	3.84	Aliexpress	Steel

## 5 Build instructions

### 5.1 Material acquisition

#### 5.1.1 Availability in the region

All the hardware components necessary for the construction of the APdrone were acquired through the AliExpress platform. These are general-use components and do not require special licenses or permits. In the context of Peru, there were no customs restrictions. Additionally, due to free trade with China, no tariffs were applied. It is important to mention that the purchase links may vary over time, and it cannot be guaranteed that the sellers will remain active in the future. For this reason, a detailed copy of the items used has been provided in the repository, complementing the information presented in the materials table. It should be noted that the delivery time may range from 3 to 4 weeks. Upon receiving all components, the assembly of the drone will proceed, following a series of instructions and recommendations to avoid damage to the components.

### 5.2 Battery duration calculation

Achieving energy efficiency is crucial to optimizing drone performance and capabilities, such as flight duration, payload capacity, and range. Several researchers are investigating multiple strategies to achieve this [22]. The calculation in this case is addressed in two scenarios: operation at maximum power and an average usage regime.

#### 5.2.1 Maximum power consumption

Table 1 consolidates the current, voltage, and power values for each component when the drone operates at 100% of its capacity [23].

With the use of a 22.2 Wh battery, an approximate flight time of 3 min 53 s is obtained (Table 2).

**Table 1:** Estimated consumption at maximum power.

Designator	Component	Current (A)	Voltage (V)	Power (W)	Notes
P10	Controller	0.1	5	0.5	*Includes sensors*
P09	ESC (4-in-1)	0.5	16	8.0	*100% operation*
P03	Motors (4x)	20.1	16	321.6	*Maximum sustained*
P06	Receiver (Rx)	0.02	5	0.1	*2.4 GHz protocol*
P13	FPV camera	0.095	12	1.14	*Normal conditions*
P11	GPS	0.15	5	0.75	*Maximum connection*
P12	VTX	1.2	9	10.8	*2.5 W output*

**Total estimated power**				**342.89 W**	

**Table 2:** Estimated flight time at maximum power.

Battery (Wh)	Flight time (h)	Minutes	Seconds	Total
22.2	0.0647	3.88	53.08	3 min 53 s

#### 5.2.2 Average power consumption

In a more realistic scenario, the motors average currents close to 9.4 A, and the *VTX* does not operate at its maximum power. Table 3 presents the estimated consumptions, significantly reducing the total power and, consequently, increasing the flight time.

Under these conditions, autonomy increases to approximately 8 min 9 s (Table 4). This type of operation better reflects the typical behavior during most missions, where phases of high power alternate with periods of hovering or less demanding maneuvers.

**Table 3:** Estimated consumption at average power level.

Designator	Component	Current (A)	Voltage (V)	Power (W)	Notes
P10	Controller	0.1	5	0.5	*Includes sensors*
P09	ESC (4-in-1)	0.5	16	8.0	*50% usage*
P03	Motors (4x)	9.43	16	150.88	*Sustained consumption*
P06	Receiver (Rx)	0.02	5	0.1	*2.4 GHz protocol*
P13	FPV Camera	0.095	12	1.14	*Normal conditions*
P11	GPS	0.15	5	0.75	*6 Satellites*
P12	VTX	0.2	9	1.8	*200 mW power*

**Total estimated power**				**163.17 W**	

**Table 4:** Estimated flight time at average power.

Battery (Wh)	Flight time (h)	Minutes	Seconds	Total
22.2	0.1360	8.16	9.80	8 min 9 s

### 5.3 Assembly

#### 5.3.1 Recommendations before assembly

The following should be considered to ensure the integrity of the components and operators: the video transmitter (P12) is highly sensitive. It is crucial to connect the power to the board only after the antenna has been properly installed. Otherwise, overvoltage without the antenna can cause irreparable damage to the device. The batteries (P08) can supply up to 150 instantaneous amperes and should be handled with care. The motors (P03) do not require a specific order in their connection wires, as this is configured through the ESC firmware. The propellers (P02) should be installed as the last step in the assembly. During the drone’s pre-programming, the motors may spin at high revolutions.

**Fig. 1:**
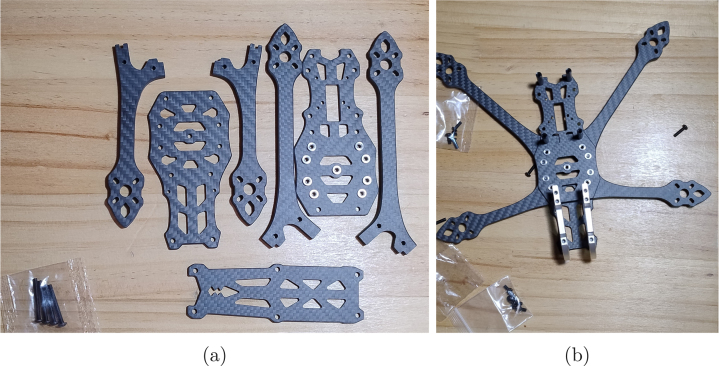
Frame assembly process: (a) structural components and (b) partially assembled frame.

#### 5.3.2 Frame assembly

The first step in assembling the APdrone is to prepare the frame. It is essential to correctly identify and organize all its parts. The frame kit (P01) includes four arms: two longer ones for the rear and two shorter ones for the front. These arms are attached to two sturdy central plates, on top of which an additional longer piece is installed at the top (see Fig. 1(a)). At this stage, it is recommended not to fully tighten this piece until all the electronics have been integrated. For more details, the complete guide is available in the (repository). A correct assembly results in a configuration similar to the one shown in Fig. 1(b).

**Fig. 2:**
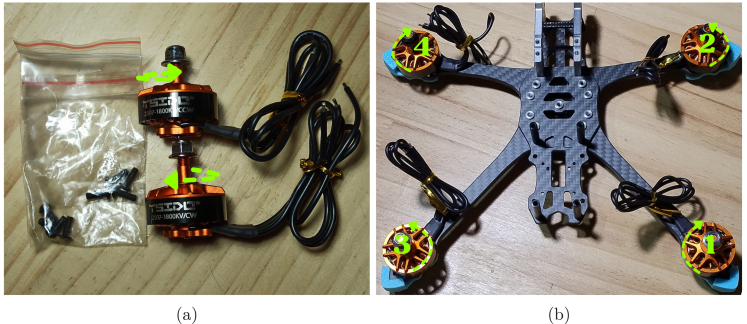
Motor types and installation: (a) view of the two types of motors CW and CCW, (b) assembled drone with motors installed without board.

Once the frame is assembled, the motors (P03) are installed, ensuring their orientation. There are two types of motors: those that rotate clockwise (*CW*) and those that rotate counterclockwise (*CCW*), as shown in Fig. 2(a). In a standard configuration, motors 1 and 4 should be *CW*, while motors 2 and 3 should be *CCW* (Fig. 2(b)). Finally, the motors are fixed to the frame using screws, ensuring they are properly secured.

#### 5.3.3 Assembly of electronic components

**Fig. 3:**
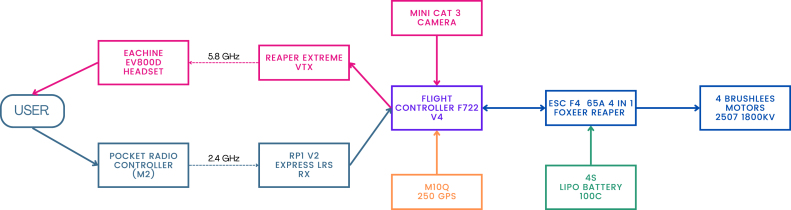
System architecture of the FPV drone: electronic components and interconnections.

**Fig. 4:**
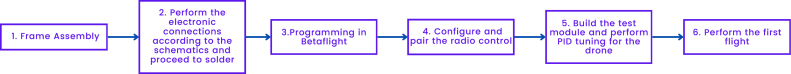
Assembly workflow for FPV drone construction.

Fig. 3 shows a diagram illustrating the interconnection of the main electronic components. And Fig. 4 provides a general overview of the assembly workflow for FPV drone construction.

The drone is controlled by the operator through the radio control (P06), which communicates with the drone via the RP1 ExpressLRS receiver (included with the radio control (P06)) at a frequency of 2.4 GHz. This receiver is connected to the flight controller board (P10). The camera (P13) is connected to this board; its signal is transmitted by the video transmitter (P12) at a frequency of 5.8 GHz. The configuration of these components is carried out using *Betaflight*, and the video signal is sent to the goggles (P05).

The flight controller is connected to the electronic speed controller (P09), which converts the control signal into power for the four brushless motors (P03). Additionally, the speed controller is connected to the battery (P08), which powers the entire drone.

The electronic part of the APdrone mainly consists of the electronic speed controller (P09), to which the battery connector and a capacitor (both included with the speed controller (P09)) are soldered in parallel, functioning as a power filter. The speed controller manages the power supplied to the motors and directs energy to the flight controller. Since the battery is a 4S (14.8 V), the speed controller reduces the voltage to the 5 V required for the flight controller (P10).

**Fig. 5:**
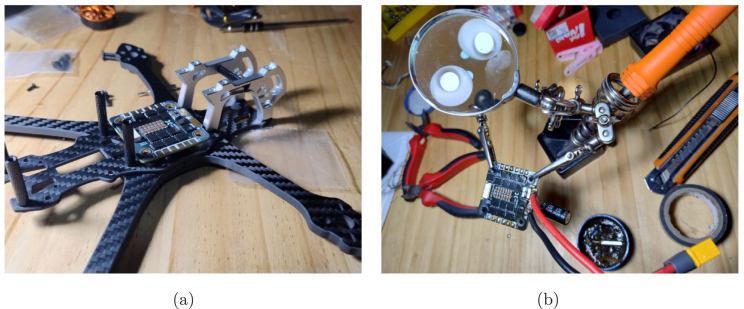
Installation of the speed controller and battery port in the APDrone: (a) variable speed drive position in the frame, (b) correct battery port installation.

**Fig. 6:**
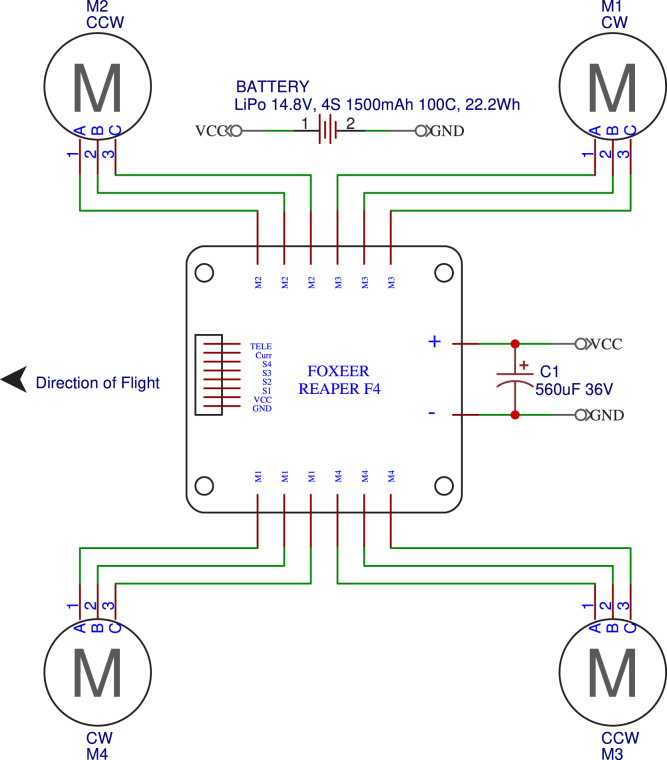
Electrical schematic of the electronic speed controller (ESC) and motor connections.

This component should be placed at the base of the central structure of the frame, isolated using silicone rubbers (included with the flight controller (P10)) to separate it from the structure to prevent interference and vibrations, as shown in Fig. 5(a). The brushless motors, battery connector, and capacitor are soldered to the speed controller, as shown in Fig. 6. The battery connector must be soldered correctly, ensuring secure connections and avoiding short circuits when connecting the battery.

For motor rotation direction, the order of the motor wire connections shown in the diagram in Fig. 6 is important. However, if after performing the motor direction test (Section 5.4.5) any motor spins in the wrong direction, the rotation can be changed via software (Section 5.4.6) without the need to re-solder.

**Fig. 7:**
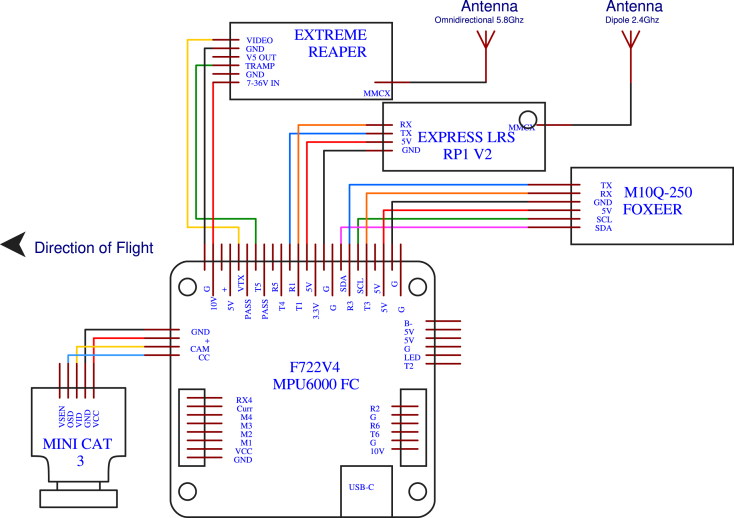
Electrical schematic of the flight controller and its connected components.

Once the assembly of the speed controller is completed, we proceed to connect the components to the flight controller in accordance with the diagram in Fig. 7. The specific steps for making these connections are detailed below:

• *Camera and OSD Connection:* First, we will connect the camera, which is equipped with a connector featuring loose wires for soldering to the OSD of the flight controller. The *OSD, VID, GND*, and *VCC* pins of the camera are soldered to the *CC, CAM, VCC*, and *GND* pins of the board, respectively, as shown in Fig. 7.
• *GPS Connection:* Next, we will connect the GPS module (P11), which is provided with a connector featuring loose wires for soldering to a serial port of the flight controller. The *TX, RX, GND, 5 V, SCL*, and *SDA* pins of the GPS are soldered to the *R3, T3, G, 5 V, SCL*, and *SDA* pins of the board, respectively, as shown in Fig. 7.
• *Control Receiver Connection:* The *ExpressLRS RP1 V2* control receiver (P06) comes with four loose wires for soldering to the receiver and a serial port of the flight controller. The *RX, TX, 5V*, and *GND* pins of the GPS are soldered to the *T1, R1, 5V*, and *G* pins of the board, respectively, as shown in Fig. 7.
• *Video Transmitter Connection:* Finally, we will connect the video transmitter (P12), which is equipped with a connector featuring loose wires for soldering to the flight controller. The *VIDEO, GND, TRAMP*, and *7-36V IN* pins of the video transmitter are soldered to the *VTX, G, T5*, and *10V* pins of the board, respectively, as shown in Fig. 7.

**Fig. 8:**
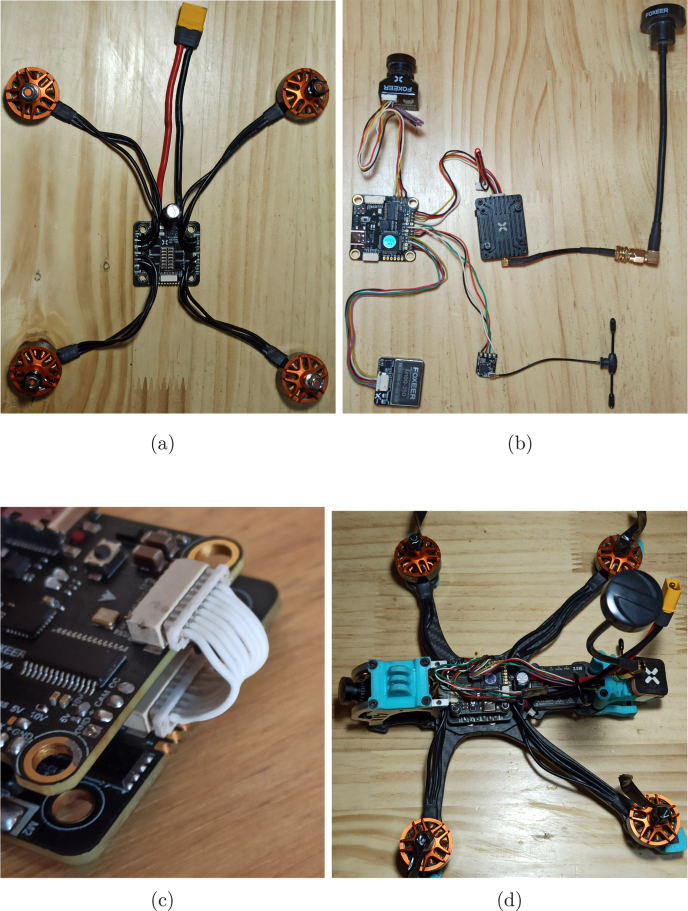
Final connection: (a) speed controller connection, (b) flight controller connection, (c) connection between boards, (d) final connection in the frame.

Following the connection diagram specified in Fig. 7, once all electronic components are installed and connected, the connection between the board and the speed controller is made with an 8-pin connector (included with the flight controller), as shown in Fig. 8. The complete connection diagram is uploaded to our repository in the file *Hardware Connection Diagram*.

### 5.4 Programming

First, download and install Betaflight Configurator 10.10.0 [24]. Before starting the configuration in Betaflight, the compatibility of the flight controller with the software was verified. In this project, the Foxeer F722 V4 flight controller (P10) is used, which is fully compatible with Betaflight, ensuring seamless integration and optimal performance. Betaflight, known for its performance, precision, advanced features, reliability, and hardware support, is considered the world’s leading multirotor flight control software, primarily preferred by FPV drone racing and freestyle communities [25].

**Fig. 9:**
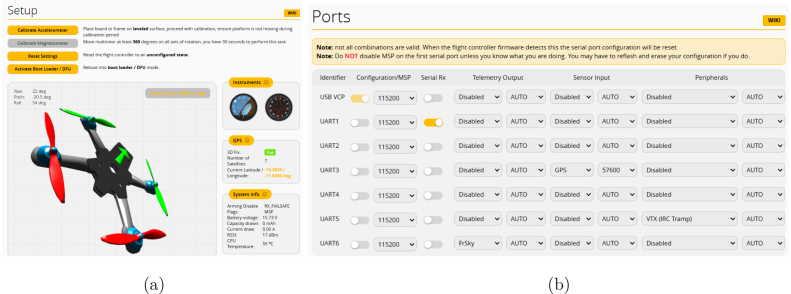
Betaflight configuration, showing (a) the system setup, accelerometer and (b) serial port configuration.

#### 5.4.1 Configuration process in betaflight

When starting Betaflight, the software automatically detects the flight controller (P10). The configuration process is carried out in several stages [26]:

1. *Accelerometer calibration:* Calibrate the accelerometer to ensure accurate drone stabilization. To perform this calibration, connect the drone via a USB Type-C cable, place it on a leveled surface, and click on the “Calibrate” button, as shown in Fig. 9(a). Ensure the drone remains stationary until the calibration process is complete.
2. *Magnetometer calibration:* Not all flight controllers support this sensor; it must be enabled while the battery is connected. Our flight controller includes a magnetometer. Calibration requires rotating the drone at least 360 degrees along all axes for approximately 30 s to ensure proper sensor alignment.
3. *Serial ports configuration:* Configure the serial ports for video transmission, remote control connection, and GPS integration (P11), as shown in Fig. 9(b). Use the electrical connection diagram as a guide Fig. 7.

#### 5.4.2 Receiver and flight modes configuration

**Fig. 10:**
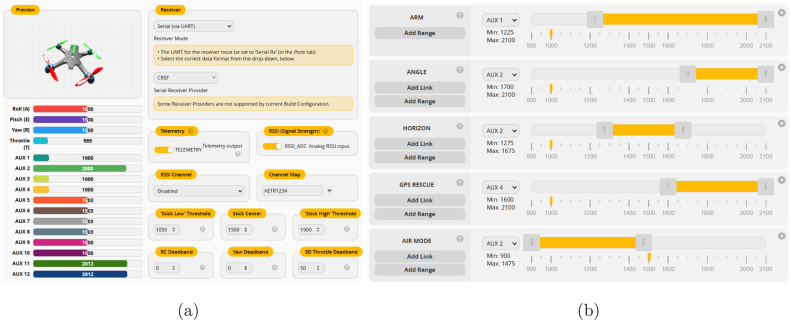
Configuration in Betaflight showing (a) the receiver setup and (b) the flight modes with their auxiliary channel assignments.

*Receiver:* Select UART as the communication method for the receiver. Choose the CRSF (Crossfire) protocol and verify the link by performing a Channel Mapping using the AETR1234 configuration, where: Channel 1 (A): Roll control, Channel 2 (E): Pitch control, Channel 3 (T): Throttle control, Channel 4 (R): Yaw control, as shown in Fig. 10(a). *Flight Mode Configuration:* Once the radio control is bound, define the flight modes and the necessary auxiliary controls for arming/disarming the drone: Angle mode, Acro mode, and Air mode [26]. Additionally, configure an auxiliary channel for arming and disarming the drone, as shown in Fig. 10(b).

#### 5.4.3 GPS configuration and real-time positioning

**Fig. 11:**
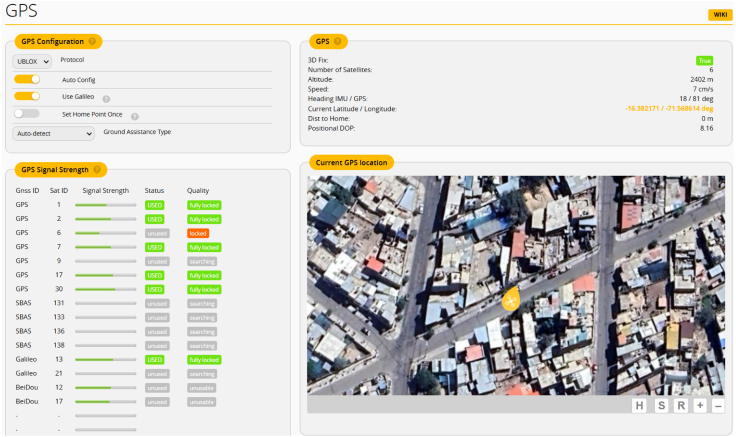
GPS module configuration interface display.

To correctly use the GPS module, we need to use *UBLOX* protocol, with the *Auto Config* and *Use Galileo* options enabled. This configuration allows integration of multiple satellite constellations, see Fig. 11.

#### 5.4.4 Video transmission and on-screen display (OSD) configuration

**Fig. 12:**
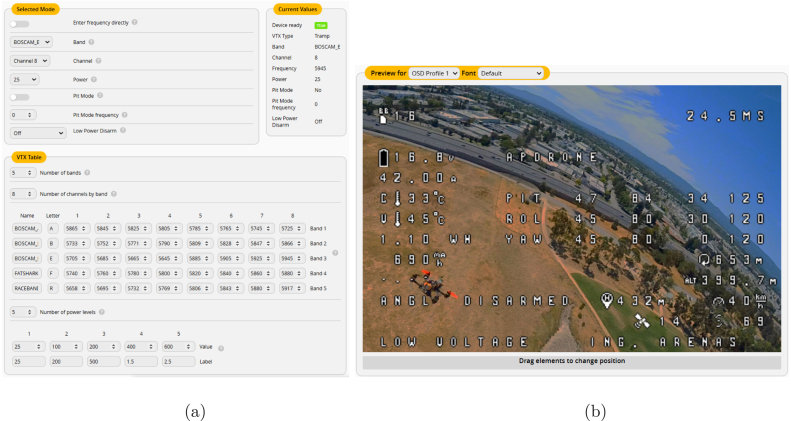
Betaflight interface showing (a) the video transmitter (VTx) frequency table configuration and (b) the on-screen display (OSD) settings.

For video transmission, assign the appropriate frequency band according to the regulations of the country of operation. In this project, the frequency of *5.945 GHz* was selected, as shown in Fig. 12(a). Betaflight OSD allows the pilot to visualize critical flight information. Relevant OSD elements were configured for the tests, such as: *battery level, instantaneous current, power, voltage, speed, etc.*, as shown in Fig. 12(b).

#### 5.4.5 Motor configuration and PID tuning

**Fig. 13:**
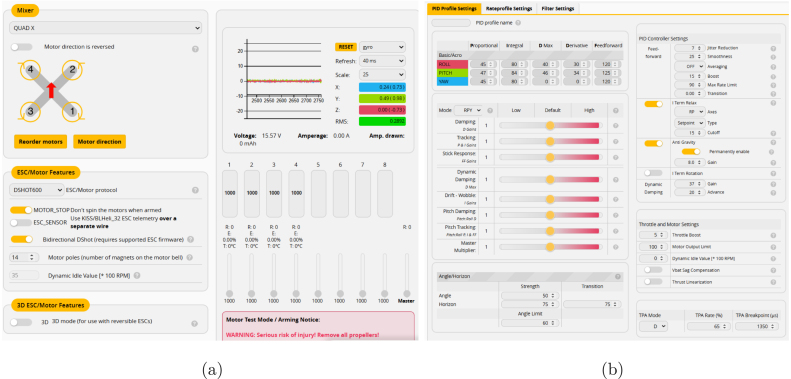
Betaflight configuration showing (a) the motor and ESC setup, and (b) the PID parameter tuning interface.

Proper motor configuration is essential to prevent imbalances and ensure stable flight performance. In a standard Quad X configuration, motors 1 and 4 rotate clockwise (CW), while motors 2 and 3 rotate counterclockwise (CCW) [27], as shown in Fig. 13(a). Once the motor setup is complete, the next step is PID tuning (Proportional, Integral, and Derivative parameters). Initially, it is recommended to start with Betaflight’s default values [20], as shown in Fig. 13(b). Fine-tuning these parameters enhances flight stability, responsiveness, and overall performance. Refer to Section 5.6.2 for further details on the tuning process.

#### 5.4.6 Forcing motor rotation direction in BLHeli

**Fig. 14:**
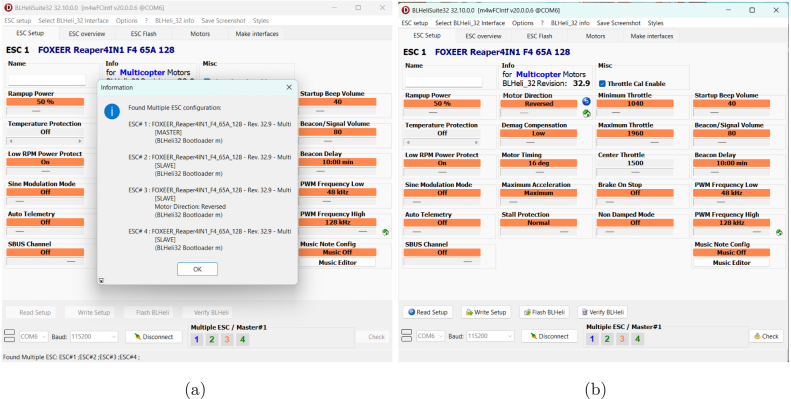
Motor configuration process using BLHeliSuite: (a) establishing the connection and (b) adjusting motor rotation direction.

If the motor rotation direction does not match the expected configuration in Betaflight, it is necessary to manually adjust the motor direction using BLHeliSuite [28]. This software allows modifying the rotation direction of any of the four motors. First, the drone must be connected to the PC using a USB cable. Then, once the program is open, the battery is connected to the drone, and the connection is verified. The motor to be modified is selected according to the distribution given in Betaflight, and we navigate to the “motor direction” option, where “reversed” is set. Finally, the changes are written to the drone using the “write setup” option [29], as shown in Fig. 14, Fig. 14.

**Fig. 15:**
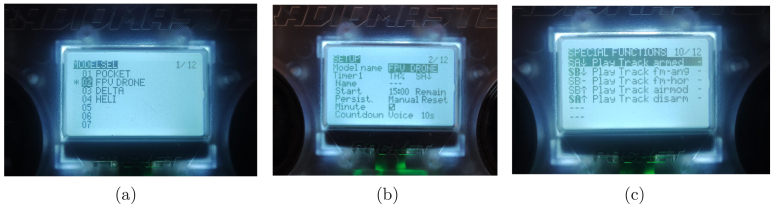
Radio controller configuration showing (a) the M2 model profile selection, (b) timer setup with a 15 min duration, and (c) assignment of voice alerts to buttons in the special functions.

### 5.5 Radio controller configuration and pairing

The radio control (P06) is equipped with the EdgeTX firmware, which is configured directly on the device. Below are the essential steps to configure and pair the radio control with the APdrone:

1. *Model selection:* Press the *MDL* button to access the available models. Select the *FPV Drone* option. Confirm the selection when an asterisk (*) appears next to the selected model (see Fig. 15(a)).
2. *Timer configuration:* Press the *PAG*> button to access the *SETUP* menu. Scroll down and select *Timer1*. Change the configuration to *TH%* to start a countdown. Assign the *SA*↓ button to start the timer. Set the time limit in minutes and change *Silence* to *Voice* to enable audible alerts. Press *RTN* to return to the main screen (see Fig. 15(b)).
3. *Voice assignment in “Special Functions”:* Navigate to page 10 *Special Functions*. Select a blank option to assign a voice to a button. Press the desired button and confirm the selection. Change the action to *PlayTrack*. Choose the specific voice (e.g., *Armed*, *Disarmed*, *Flightmode*) to assign to the button (see Fig. 15(c)).

#### 5.5.1 Pairing the radio controller with the drone

**Fig. 16:**
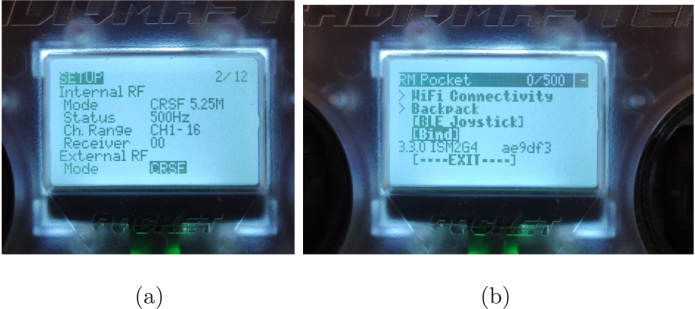
Radio controller pairing process showing (a) the selection of CRSF mode in the external RF settings and (b) the pairing procedure with the drone.

To bind the radio controller with the APdrone, follow the steps below:

1. *Activating internal RF communication:* Press *MDL* to select the configured model. Press *PAG*> to go to page 2. Scroll to *Internal RF* and change it from “OFF” to “CRSF” (see Fig. 16(a)).
2. *Bind mode:* Activate *Bind* mode by connecting the drone’s battery twice quickly and keeping the last connection (see Fig. 16(b)).
3. *Configuration on the radio controller:* Press the *SYS* button on the radio controller. Select *ExpressLRS* from the menu. Navigate to the *Bind* option and select it to start the pairing process.
4. *Completing the Pairing:* Ensure the left joystick (throttle) is fully down before arming the drone. Arm the drone and slowly move the joystick upward to start the motors.

### 5.6 PID tuning procedure

#### 5.6.1 Constraints of model-based PID tuning

Although mathematical models are useful for understanding system dynamics and optimizing parameters under ideal conditions, their applicability is limited due to simplifications that do not account for factors such as vibrations, wear, or external disturbances [30], [31].

Considering the advantages and disadvantages outlined in Table 5, this project adopts an empirical approach using Blackbox Explorer, which enables practical, replicable, and hardware-specific adjustments for the drone based on its sensor data.

**Table 5:** Comparison between mathematical models and empirical tuning for PID control.

Aspect	Mathematical models	Empirical tuning (Blackbox explorer)
Advantages	Deep understanding of the system. Theoretical prediction under ideal conditions. Optimization without test flights.	Real flight data logging. Hardware-specific adjustments. Fast and replicable iterations.

Disadvantages	Not universally applicable. Ignores real-world factors. High complexity in design and execution. Dependent on the quality and accuracy of the mathematical model. Requires expertise in advanced control techniques. Computational time needed for simulations.	Requires test flights and technical analysis. Results sensitive to environmental variations.

#### 5.6.2 PID tuning procedure in betaflight

In the *Betaflight firmware*, the PID controller is implemented in discrete form to operate on resource-limited microcontrollers at a typical frequency of 4 kHz. This controller extends the capabilities of a PD controller by including an integrator term, specifically designed to reduce steady-state error by considering the system’s history and correcting accumulated errors [32]. While this term improves performance against persistent errors, it also introduces an additional integrator that can cause instability if not properly tuned.

According to our analysis of the Betaflight source code, each axis (*Roll*, *Pitch*, and *Yaw*) is regulated through the iterative calculation of Proportional, Integral, and Derivative (PID) outputs. However, if necessary, complementary terms can be applied to enhance stability and responsiveness, including:

• *Feedforward (F)*: Anticipates abrupt variations in the *setpoint*, reducing response latency.
• *Throttle PID Attenuation (TPA)*: Reduces PID gains when the throttle approaches its maximum level, avoiding excessively sharp reactions.
• *I-term Limitation*: Prevents excessive accumulation of the integral term (*windup*), mitigating the risk of instability.

**Fig. 17:**
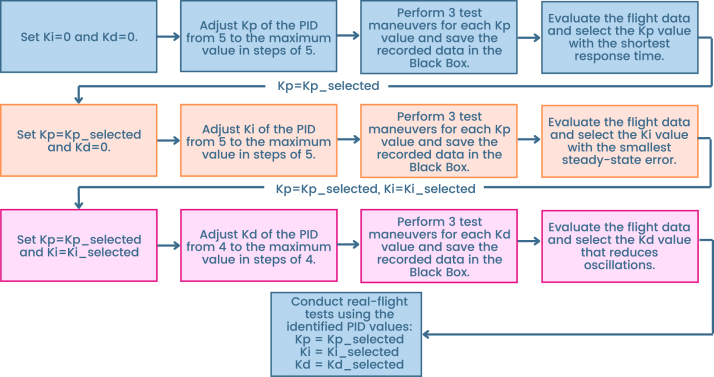
Step-by-step PID tuning workflow using blackbox explorer.

Based on these findings, it was determined that an empirical approach is more practical for tuning the PID constants on the Roll, Pitch, and Yaw axes. The general tuning procedure, which uses *Blackbox Explorer* to collect and analyze flight data, is illustrated in Fig. 17.

The maximum values were those permitted by the Betaflight platform and correspond to safe operational limits intended to prevent overloading of the controller and ensure flight stability. In this study, the selected maximum values were: for the roll axis, $K_{p}$= 90, $K_{i}$ = 120, $K_{d}$ = 40; for the pitch axis, $K_{p}$ = 185, $K_{i}$ = 200, $K_{d}$ = 40; and for the yaw axis, $K_{p}$ = 165, $K_{i}$ = 150, $K_{d}$ = 65. The specific conditions and parameters of the test maneuvers are presented in Table 6.

#### 5.6.3 Test platform for drone evaluation

**Fig. 18:**
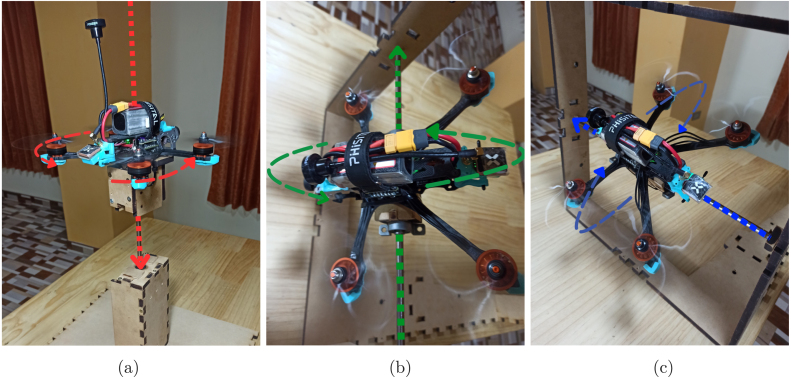
Design of experimental platforms for (a) Yaw, (b) Pitch, and (c) Roll axis evaluation.

Although the $K_{p}$, $K_{i}$, and $K_{d}$ parameters can be adjusted directly during flight, this practice poses significant risks, particularly for inexperienced users. Improper tuning may lead to control board failures, severe structural damage from crashes, or even hazards to people and property. Therefore, a controlled testing environment is essential for safely and effectively evaluating PID coefficients. To address this, two dedicated test platforms were designed and built to analyze the drone’s behavior under controlled conditions. As illustrated in Fig. 18, these platforms consist of MDF boards and 8 mm metal rods with linear bearings to minimize friction. This setup securely mounts the drone while allowing free rotation along each axis with minimal resistance, ensuring both precise measurements and operator safety. Videos demonstrating the test platforms’ functionality, along with their CAD design files, are available in our repository under the *Supplementary Data/Multimedia Evidence of Test Platform and Functionality* folder.

**Fig. 19:**
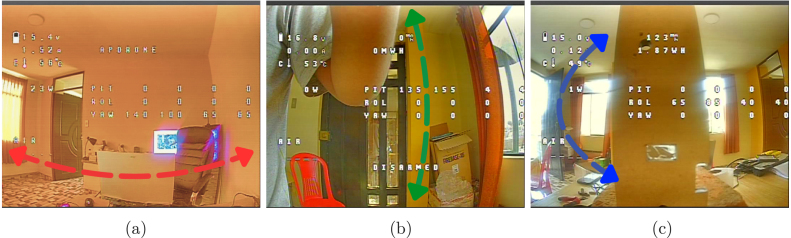
(a) Yaw, (b) Pitch, and (c) Roll axis rotations captured from the drone camera on the test platform.

The platforms provide freedom of movement for each axis of interest. The structure shown in Fig. 18(a) allows rotation on the *Yaw* axis, while those shown in Fig. 18, Fig. 18 enable rotation on the *Pitch* and *Roll* axes, respectively. As seen in Fig. 19, we provide a perspective from the drone’s camera, confirming the platform’s functionality by replicating the maneuvers associated with each axis in a controlled manner. Thus, the PID coefficient tuning process (see Fig. 17) is conducted safely and in a controlled environment, preserving the integrity of both the equipment and its surroundings.

To ensure the validity of the tuning process, the influence of the test platforms’ moment of inertia on the drone’s dynamics was analyzed. The results of this analysis show that the inertia of the platforms is less than 10% of the drone’s total inertia for the corresponding axes of rotation (9.22% for the Yaw axis and 6.78% for the Pitch/Roll axes). A summary of these calculations is presented in Appendix G.

#### 5.6.4 Characterization of test maneuvers for PID tuning

Each parameter of the PID controller ($K_{p}$, $K_{i}$, $K_{d}$) evaluates different aspects of control, so specific maneuvers were designed to expose and isolate the particular characteristics of each coefficient, maintaining test conditions as similar as possible to facilitate comparison. The proportional term ($K_{p}$) determines the drone’s reaction speed to the setpoint signal: a very high value results in excessively sensitive movements, while a very low value causes a slow response, leading in both cases to errors between the desired angular velocity and that measured by the gyroscope. The integral constant ($K_{i}$) influences the drone’s ability to maintain stability in the face of steady-state disturbances: if it is too high, it can cause stiffness and low-frequency oscillations, while an insufficient magnitude makes it difficult to maintain the desired angle. Finally, the derivative constant ($K_{d}$) mitigates peaks generated by the other terms in both transient and steady states; however, excessively high values can introduce high-frequency noise and overload the motors, while very low values result in bouncing during final maneuvers. Table 6 summarizes the specific characteristics of the maneuvers designed to evaluate the behavior of the PID controller.

**Table 6:** Characterization of test maneuvers for PID tuning.

Axis	Proportional ($K_{p}$)	Integral ($K_{i}$)	Derivative ($K_{d}$)
*Roll* and *Pitch*	Drone rotation from clockwise to counterclockwise, alternating between four fast and four slow cycles. Duration: 12 s. The analysis focuses on the transient response (setpoint≠0°/s).	Drone rotation maintaining between +45° and −45° for four cycles. Duration: 14 s. The analysis focuses on the steady-state (setpoint=0°/s).	Drone rotation from clockwise to counterclockwise, maintaining between +45° and −45° for four cycles. Duration: 8 s. The analysis includes both transient and steady-state responses.

*Yaw*	Same as Roll and Pitch.	Execution of four movements (two clockwise and two counterclockwise) with a constant opposing disturbance force. Duration: 10 s. The analysis focuses on the steady-state (setpoint=0°/s).	Rapid rotation clockwise and counterclockwise for four cycles, with a constant opposing disturbance force. Duration: 6 s. The analysis includes both transient and steady-state responses.

#### 5.6.5 Python-based data processing for blackbox PID tuning

**Fig. 20:**
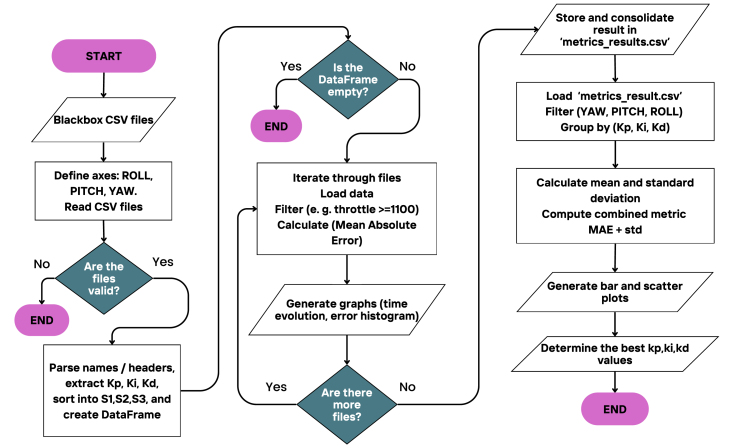
Flowchart of data processing for PID tuning.

The data obtained from each test are stored in the drone’s *blackbox*, which has a capacity of 16 MB and generates files in BBL format. For analysis, the online tool *Blackbox Explorer* [21] is used, allowing the selection of the desired flight log and exporting the information to a CSV file. There is a video in our repository showing the steps. Subsequently, these data are processed using a program developed in *Python*, specifically created for this task. This program iteratively implements the algorithm described in the previous section, adapting to the large amount of data generated by the *blackbox*. The overall process is simplified in the flowchart shown in Fig. 20.

As shown in Fig. 20, the program analyzes the data using the *Mean Absolute Error* (*MAE*), which calculates the average magnitude of the absolute errors between the predicted and actual values [33]. To ensure the reliability of the results, each coefficient configuration is subjected to three independent tests ($S_{1}$, $S_{2}$, and $S_{3}$), as described in Fig. 17. Specific details of the processing are provided in Appendix D. Based on the *MAE* values obtained, the mean and standard deviation of each test series are calculated. The coefficient configuration with the lowest average *MAE* and the smallest standard deviation is prioritized, thus ensuring optimal tuning of the PID controller.

### 5.7 Empirical optimization of PID gains using MAE analysis

**Fig. 21:**
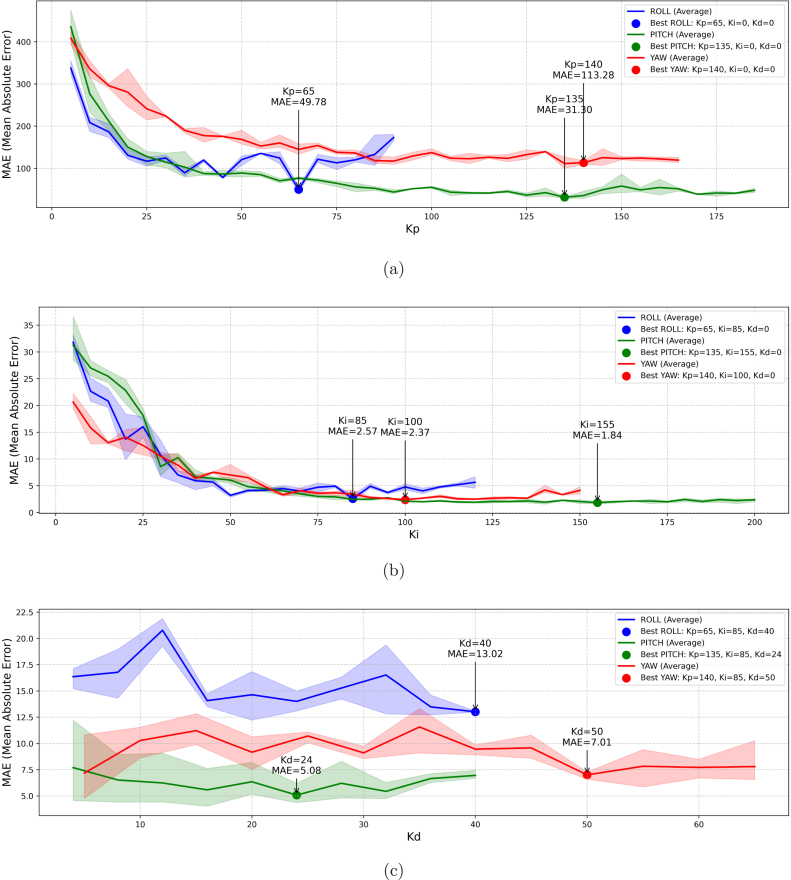
MAE-based tuning of $K_{p}$, $K_{i}$, and $K_{d}$ for the *Roll*, *Pitch*, and *Yaw* axes. (a) Transient response error reduction: $K_{p}$ MAE analysis. (b) Steady-state error reduction: $K_{i}$ MAE analysis. (c) Oscillation damping evaluation: $K_{d}$ MAE analysis. (For interpretation of the references to color in this figure legend, the reader is referred to the web version of this article.)

Based on the experimentation with the designed maneuvers and the data processing in *Python*, the $K_{p}$ value that minimizes the *MAE* was determined for each axis. As illustrated in Fig. 21(a), once this value was identified, the procedure described in Fig. 17 was followed to adjust $K_{i}$ (Fig. 21(b)) and finally $K_{d}$ (Fig. 21(c)).

Fig. 21 shows the variation of the *MAE* as a function of each PID controller coefficient for the three axes: *Yaw* (red line), *Roll* (blue line), and *Pitch* (green line). The shaded areas indicate the minimum and maximum values obtained in three independent tests ($S_{1}$, $S_{2}$, and $S_{3}$), while the central curve represents the average of those measurements. Based on these results, the control coefficients ($K_{p}$, $K_{i}$, $K_{d}$) and the associated *MAE* for each were identified and are summarized in Table 7.

Table 7 presents a summary of the results from the PID control tuning procedure. According to our methodology outlined in Section 5.6.2, the $K_{p}$ value was first determined by comparing the calculated MAE value based on the maneuver criteria specified in Table 6. With this value applied to the controller, the $K_{i}$ coefficient was then obtained, followed by the $K_{d}$ coefficient, for each axis. Since each coefficient ($K_{p}$, $K_{i}$, $K_{d}$) and each axis (roll, pitch, yaw) has its own criteria (Table 6), it is expected that the MAE value varies significantly between each axis and each coefficient.

**Table 7:** PID tuning results.

Axis	Proportional		Proportional and Integral			Proportional, Integral and Derivative			
	$K_{p}$	MAE	$K_{p}$	$K_{i}$	MAE	$K_{p}$	$K_{i}$	$K_{d}$	MAE
Roll	65	49.78	65	85	2.57	65	85	40	13.02
Pitch	135	31.30	135	155	1.84	135	155	24	4.08
Yaw	140	113.28	140	100	2.37	140	100	50	7.01

**Fig. 22:**
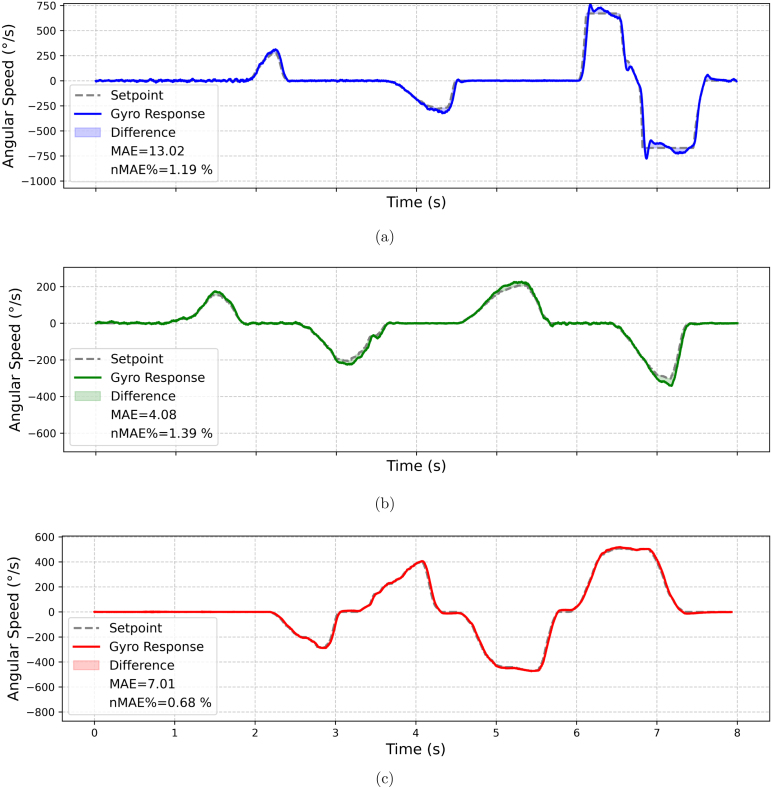
Behavior of the drone in the three axes during test maneuvers on the platform with adjusted values ($K_{p}$, $K_{i}$, $K_{d}$). Gyroscopic response vs. setpoint on the (a) Roll, (b) Pitch, and (c) Yaw axes.

Table 8 shows the coefficients of the PID controller of the 3 axes resulting from the tuning procedure. These coefficients provide stable performance, minimizing the *Mean Absolute Error* and improving the response both in the transient phase and in the steady state. Fig. 22 illustrates the drone’s behavior when performing test maneuvers on the platform with these adjusted values. To evaluate the performance of the results, the Normalized Mean Absolute Error (nMAE) will be used. This metric is employed to assess the accuracy of prediction or estimation models, particularly when comparing errors across different scales or units [34]. Details of the nMAE metric are provided in the Appendix E.

**Table 8:** PID coefficients.

Axis	Proportional ($K_{p}$)	Integral ($K_{i}$)	Derivative ($K_{d}$)
*Roll*	65	85	40
*Pitch*	135	155	24
*Yaw*	140	100	50

As shown in Fig. 22, the *Roll* axis exhibits a fast response to the *setpoint*, with slight overshoots dampened by $K_{d}$. The $K_{i}$ term corrects steady-state errors, converging to a stable state. On the *Pitch* axis, the response approaches ideal conditions, with minimal oscillations corrected by $K_{d}$ and no notable overshoots, resulting from the precise tuning of $K_{p}$. A slight steady-state error persists, which, however, does not significantly affect overall performance. Finally, on the *Yaw* axis, the drone follows the *setpoint* with precision, without overshoots or steady-state errors, achieving fast and stable control aligned with the estimated optimal $K_{p}$, $K_{i}$, and $K_{d}$ values. The legend of Fig. 22 shows the calculated nMAE value, which is notably very small.

## 6 Operating instructions

### 6.1 Software requirements

To perform all the steps leading up to this point, the following software dependencies must be installed. If these are already met, you may skip this section and proceed directly to the drone flight procedures.

1. Windows 10/11 (64-bit) – This study uses Windows 11 24H2 Home Single Language.
2. Betaflight Configurator 11.0.0.
3. BLHeliSuite Rev32.10.0.0
4. Python 3.12.5.
5. Required Python libraries:
  • matplotlib==3.10.0
  • numpy==2.2.2
  • pandas==2.2.3
  • seaborn==0.13.2
6. Enhanced Blackbox Explorer 3.7.0 (available online at https://blackbox.betaflight.com/).
7. Visual Studio Code 1.96.4.
8. Required Visual Studio Code extensions:
  • mechatroner.rainbow-csv@3.15.1
  • ms-python.debugpy@2024.14.0
  • ms-python.python@2024.22.2
  • ms-python.vscode-pylance@2024.12.1

### 6.2 Pre-flight preparation

1. *Visual Inspection*:
  • Check the physical condition of the frame, propellers, and electronic connectors.
2. *Power verification*:
  • Ensure the main battery has a minimum voltage of 12 V.
  • Confirm that the remote control has a voltage above 6 V.
3. *Power-up and calibration*:
  • Connect the battery to the drone; the controller will complete its boot sequence.
  • The system will automatically calibrate the gyroscope. The drone will be ready for takeoff once the GPS acquires a signal from at least 3 satellites.

### 6.3 Operation procedure

1. *Takeoff*:
  • Select the appropriate flight mode (angle, horizon, or acro) from the remote control, based on your level of experience.
  • Arm the drone using the designated switch.
  • Gradually increase the throttle to reach approximately 1 m of altitude.
2. *In-flight navigation*:
  • Use the right stick to control *roll* and *pitch* movements. Use the left stick for the *yaw* axis and throttle.
  • Continuously monitor the OSD system information, including battery status, altitude, and speed.
  • Keep the drone within direct line of sight for greater safety.

### 6.4 Landing and shutdown

1. Gradually lower the altitude and disarm the drone using the remote switch.
2. Disconnect the drone’s power supply first, then turn off the remote control.

## 7 Validation and characterization

A series of tests were conducted to measure its performance under real flight conditions.

### 7.1 Battery autonomy testing procedure

The battery autonomy tests were conducted under two conditions: maximum and normal power modes. The first test was performed in *Air Mode* to maintain continuous thrust at maximum power. To prevent unintended movement or takeoff, the drone was secured using the test module described in Section 5.6.3, with an additional 3 kg load. Additionally, the load capacity at maximum power is measured The normal power test was conducted in *Angle Mode* during an uninterrupted real flight, using only the drone’s weight. Each test was repeated five times to ensure consistency. These conditions were selected to simulate extreme and standard operating scenarios, providing a comprehensive analysis of battery performance for comparison with the calculations in Section 5.2.1.

**Table 9:** Battery autonomy and maximum load capacity test results for the APdrone.

Battery autonomy and load capacity			
Test	Load at maximum power (kg)	Time at maximum power (s)	Time at normal power (s)
Test 1	1.977	204.1	492.6
Test 2	1.953	190.8	489.9
Test 3	1.975	212.2	493.4
Test 4	1.957	199.3	488.5
Test 5	1.947	187.4	503.1

**Average**	**1.962**	**198.8**	**493.5**

**Fig. 23:**
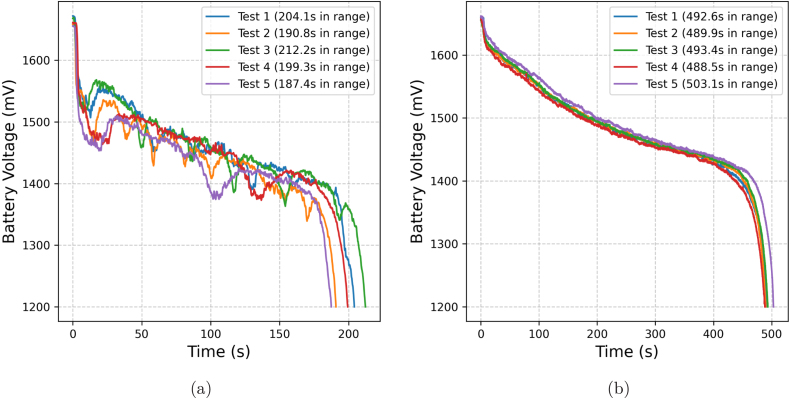
Battery discharge curves at (a) maximum and (b) normal power.

### 7.2 Results from multiple flights in urban environments

**Fig. 24:**
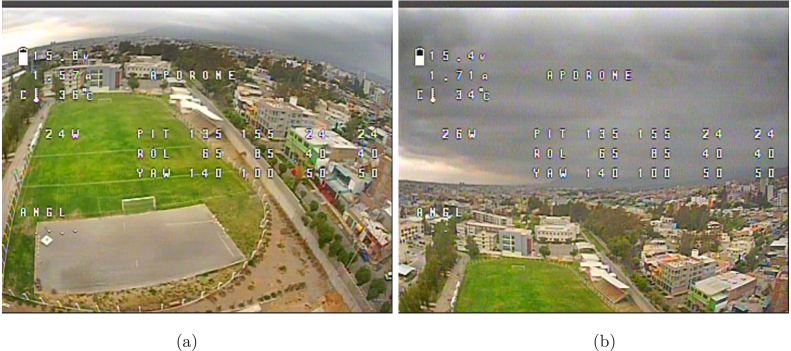
Flight in an urban environment: (a) acceleration takeoff and (b) navigation flight, viewed from the OSD.

**Fig. 25:**
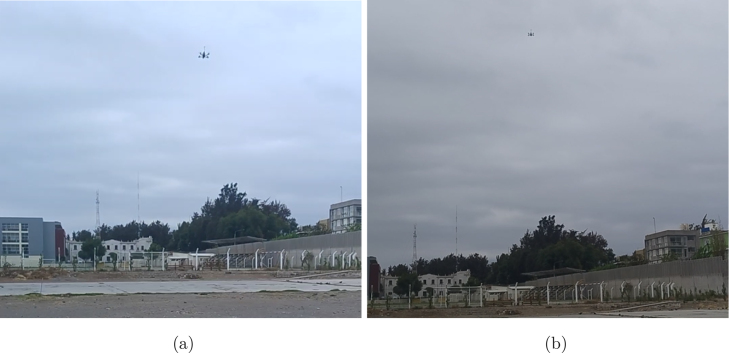
Flight in an urban environment: (a) acceleration takeoff and (b) navigation flight, viewed from the ground.

Five test flights were conducted in an urban environment, maintaining similar environmental conditions (Figs. 24(a), 24(b), Fig. 25, Fig. 25). During each flight, takeoff and landing cycles were performed, and data were recorded using the APdrone’s *blackbox*. Subsequently, the data were extracted and analyzed from the file generated by this system. The most relevant parameters are presented in Table 10.

**Table 10:** Results of multiple APdrone flights in urban environments.

Parameter	Units	Flight 1	Flight 2	Flight 3	Flight 4	Flight 5
Flight conditions						

Average altitude	m	4.85	1.63	1.69	4.12	2.08
Ambient temperature	K	290.15	291.15	291.15	290.15	290.15
Relative humidity	%	69	66	66	67	68
Wind speed	m/s	2.78	3.05	3.61	3.61	3.61

Drone performance						

Initial voltage	V	16.37	16.67	15.75	15.05	15.31
Final voltage	V	15.08	15.29	14.90	14.41	14.54
Flight time (OSD)	s	145	182	245	258	256
Average instantaneous consumption	W	23.35	23.24	25.03	25.21	26.80
Maximum instantaneous consumption	W	211.69	183.20	251.10	196.17	180.49
Average speed	m/s	3.26	3.84	1.98	2.34	1.65
Maximum speed	m/s	3.60	3.80	4.28	3.60	3.15

Other parameters						

Number of connected GPS satellites	N/A	5	4	3	6	5
Maximum altitude reached	m	14.74	5.62	4.37	5.10	2.08

### 7.3 Flight results in open field environment

**Fig. 26:**
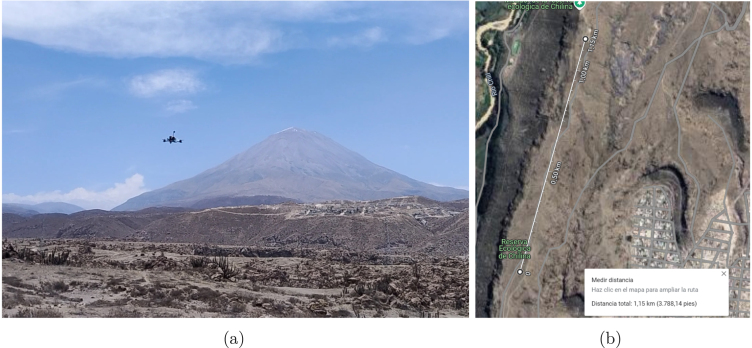
Flight in Open-Field environment: (a) field flight test and (b) test distance.

To maximize performance testing, experimental flights were conducted in a larger area away from the city, as shown in Fig. 26, Fig. 26. This open space allowed for the execution of more complex maneuvers and longer flight distances safely. The results obtained are detailed in Table 11.

**Table 11:** Results of APdrone flights in open-field environments.

Parameter	Units	Flight 1	Flight 2	Flight 3	Flight 4	Flight 5
Flight conditions						

Average altitude	m	2.64	1.91	2.65	17.25	0.74
Ambient temperature	K	296.15	296.15	296.15	295.15	293.15
Relative humidity	%	39	39	39	41	44
Wind speed	m/s	4.45	5	5	4.72	5

Drone performance						

Initial voltage	V	16.14	16.47	16.58	15.08	15.48
Final voltage	V	15.12	16.15	15.88	14.44	15.05
Flight time (OSD)	s	28	17	83	14	19
Average instantaneous consumption	W	29.24	31.08	28.67	32.25	24.56
Maximum instantaneous consumption	W	266.99	80.05	61.07	244.87	163.68
Average speed	m/s	3.13	5.70	1.83	2.93	2.85
Maximum speed	m/s	16.02	18.71	5.75	6.60	8.25

Other parameters						

Number of connected GPS satellites	N/A	23	18	29	28	26
Maximum altitude reached	m	10.4	9.91	7.73	30.56	6.12

**Table 12:** Speed results.

Parameter	Units	Flight 1	Flight 2
Acceleration time 0–16.66 m/s	s	3.4	2.0

### 7.4 Analysis of results

The experimental results confirm that the APdrone achieves an average flight time of 8 min and 12 s under standard operating conditions (Table 9). This duration exceeds the theoretical flight time estimate by 0.6% (Table 4). Under maximum power conditions, the recorded flight time ranges from 3 min and 18 s, as shown in Table 9. This performance deviates by -15% from the theoretical expectations derived from power consumption models (Table 2). Fig. 23 illustrates the battery discharge behavior under two distinct operating conditions. In the (Fig. 23(a)) that shows the discharge curve at maximum power, the voltage experiences a rapid 10% drop within the first 10 s, attributed to the high instantaneous power demand. Additionally, the discharge rate remains irregular, likely influenced by transient current spikes. Conversely, in the (Fig. 23(b)) that shows the discharge curve at normal power, the voltage decrease is more gradual and stable, with only a 3.5% drop in the first 10 s. The discharge profiles exhibit a consistent trend across all trials, confirming the expected battery behavior. These findings emphasize that continuous operation at 100% power output is not advisable due to the significantly increased discharge rate. Prolonged operation under such conditions could severely degrade the battery lifespan and reduce overall flight efficiency.

Tests in urban environments (Table 10) show that the drone maintains good satellite reception (between 3 and 6) and performs well in moderate winds of 2.78 m/s. During short flights of around 200 s, the battery voltage decreases by 1 V from its initial value, a very good linear discharge. However, when the drone executes quick direction changes or suddenly increases its speed, instantaneous consumption can exceed 210 W. Even so, the average consumption remains around 25 W. With a total weight of 628.4 g, the APdrone achieves speeds in the range of 1.94–4.16 m/s, which is considered sufficient for agile movements in urban areas. This test was conducted under relative cold weather conditions (290.15 K); however, performance in high-temperature environments has not been evaluated. It is know that components such as motors, the video transmitter, and the battery exhibit temperature-dependent performance variations, which could impact flight endurance and overall system stability.

In open-field tests (Table 11), the drone demonstrated its ability to fly in winds of up to 5 m/s, although some lateral displacement was observed due to gusts. This effect, however, can be manually compensated by the pilot. The average power consumption is approximately 30 W, with peaks of up to 266 W during sudden acceleration or ascent maneuvers. Additionally, due to reduced electromagnetic congestion in rural areas, between 18 and 20 GPS satellites were recorded, enhancing navigation accuracy.

This test was conducted under an ambient temperature of 296.15 K. Finally, Table 12 compiles the speed test results. It was confirmed that the drone can reach 16.66 m/s in just 2–3 s, demonstrating its ability for rapid acceleration when higher power is required.

### 7.5 Validation of PID tuning

The validation of the tuning method applied to the FPV drone is a fundamental aspect of this project. To achieve this, one of the test flights conducted in an open field was selected, during which the pilot executed simultaneous maneuvers involving all three control axes. This environment provided the necessary space for larger amplitude maneuvers.

**Fig. 27:**
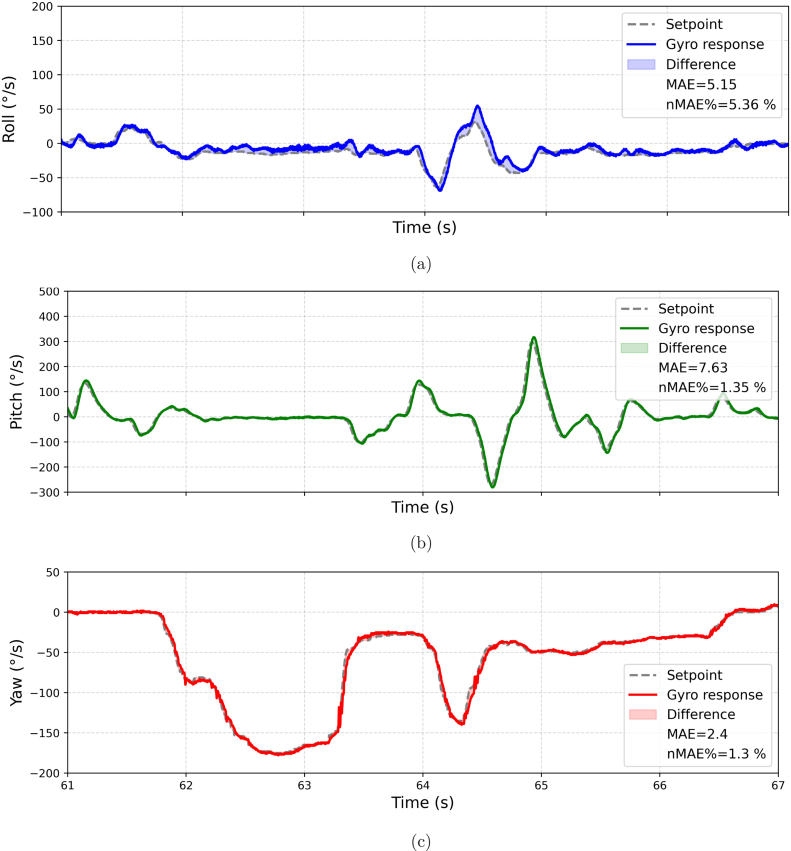
Gyroscope values vs. setpoint for the (a) *Roll*, (b) *Pitch*, and (c) *Yaw* axes in an actual flight.

Fig. 27 shows a 6-s segment of this flight, where the drone operated near a rocky formation in Arequipa. During this period, significant movements were performed on the *Roll*, *Pitch*, and *Yaw* axes to evaluate the effectiveness of the $K_{p}$, $K_{i}$, and $K_{d}$ values obtained through empirical tuning.

The Fig. 27 shows the calculated nMAE values from a real flight, slightly higher than those obtained from test platform flights. These nMAE values remain small, indicating that the actual measured value has minimal deviation from the setpoint, implying good performance and proper calibration.

As shown in Figs. 27(a), 27(b), and 27(c), the *Pitch* and *Yaw* axes exhibited particularly stable behavior, while *Roll* showed slight additional oscillations. This variation could be attributed to sudden wind gusts recorded at approximately 15 m of altitude, which caused momentary disturbances. Nevertheless, the drone successfully compensated for these variations, maintaining overall satisfactory performance.

Globally, the results of the abrupt maneuver executed on all three axes reflect effective control due to the selected $K_{p}$, $K_{i}$, and $K_{d}$ values, even under adverse external conditions. This validates the empirical tuning method used, demonstrating its suitability for FPV applications in environments with variable error factors.

### 7.6 Capabilities and limitations

**Capabilities**

• *Autonomy and Performance:* The APdrone can sustain over 8 min of continuous flight under normal operating conditions (Table 9), aligning with estimated power consumption(Table 4). Under maximum demand, while carrying a payload of 0.98 kg, flight time is reduced to just over 3 min, allowing for intense maneuvers and rapid ascents.
• *Stability and PID Control:* The empirically tuned PID control parameters ($K_{p}$, $K_{i}$, and $K_{d}$) (Fig. 22) ensure stable flight across *Roll*, *Pitch*, and *Yaw* axes, even in windy conditions (Table 10).
• *Payload Capacity and Acceleration:* With a thrust output of 1.96 kg, the drone can carry payloads up to 0.98 kg of payload while maintaining a thrust-to-weight ratio of 1.22:1. These characteristics make it ideal for applications ranging from aerial inspections or recreational activities to research and development projects [35]. It achieves an acceleration from 0 to 16.66 m/s in approximately 2 s, making it suitable for various applications.
• *Range and GPS Reception:* Field tests (Fig. 26, Table 11) confirm that the APdrone maintains stable connections with 18–23 GPS satellites and a reliable video link up to 1.15  km. Its theoretical range is significantly higher under ideal line-of-sight conditions. Additionally, the drone can be “armed” remotely from 1.15  km, thanks to its robust radio link.
• *Modular Design and Upgradeability:* Utilizing open-source software and hardware, users can customize settings, replace components, and enhance subsystems — such as increasing battery capacity — without being restricted to proprietary brands.
• *Structural Durability:* Built with a 5-inch carbon fiber frame and toroidal propellers, the drone is resistant to impacts and falls, making it ideal for high-risk testing environments.

**Limitations**

• *Payload Impact on Autonomy:* To achieve an agile thrust-to-weight ratio of 2:1 [23], which is ideal for high-performance maneuvers, the recommended maximum payload is approximately 350 g. The maximum payload significantly reduces flight time to around 3–4 min. This limits its ability to support long-duration missions involving heavy equipment such as 4K cameras or LIDAR sensors.
• *Weather Sensitivity:* Stability can be affected by strong winds exceeding 5 m/s or adverse weather conditions. At altitudes above 15 m, sudden gusts may cause disturbances in the Roll axis (Fig. 27(a)), requiring manual compensation by the pilot.
• *Real vs. Theoretical Range:* While the radio and video link theoretically exceed 1.15  km in ideal conditions, electromagnetic interference and obstacles can substantially shorten this effective range.
• *Technical Knowledge Requirement:* Assembly, PID tuning, and component configuration require expertise in FPV systems, electronics, and flight software (*Betaflight, BLHeli*). Novice users may struggle with maintenance and repairs without proper training.
• *High Power Consumption During Intense Maneuvers:* Power draw can exceed 220–255 W during rapid accelerations or headwinds, increasing the temperature of critical components such as ESCs and motors. Frequent inspections are necessary to prevent overheating.
• *Pre-Flight Checks:* Before each flight, users must verify propeller and antenna security, battery connections, and sensor calibration to prevent critical failures during operation.
• This work employs a PID-based control strategy implemented via the Betaflight platform, which remains the de facto standard in FPV racing and freestyle drone communities due to its proven reliability and ease of tuning. Alternative control strategies, including LQR, MPC, or nonlinear methods, while theoretically advantageous, were deemed unfeasible for this project due to computational limitations, firmware constraints, and the complexity of accurate dynamic modeling in real-world FPV conditions. Furthermore, advanced controllers would require substantial firmware redevelopment, hardware adaptation, and a bespoke development environment, undermining the replicability and accessibility objectives of this work.
• In this study, standard PID control was used via Betaflight, without additional features such as Feedforward, Throttle PID Attenuation, or I-term Limitation, to ensure replicability and controlled evaluation. These options, while beneficial for optimizing high-speed maneuvers, introduce pilot-dependent variability. Additionally, while in-flight PID fine-tuning represents a valuable practice in FPV applications, it was not included in this study to maintain objective, replicable conditions. Future work may explore these enhancements and performance optimization strategies under varied flight scenarios. Future work will explore the integration and benchmarking of advanced control techniques in simulation environments such as Gazebo or AirSim, which offer a controlled setting free from the hardware limitations inherent to FPV drones. This approach will enable a rigorous comparative analysis of PID control against nonlinear, adaptive, or optimal controllers while preserving the replicability and practicality emphasized in the current study.

Overall, the APdrone stands out as a versatile, high-performance platform capable of executing complex missions across diverse scenarios. Compared to similar drones in its class, such as the DJI Mavic 3 Pro and Nazgul Evoque F6 V2 O3 GPS, the APdrone offers superior customization through open-source software, a more durable carbon fiber frame, and a higher thrust-to-weight ratio, enabling aggressive flight maneuvers and increased payload capacity. Its precise PID control, mechanical robustness, and potential for hardware and software upgrades make it ideal for aerial inspections, research, and development projects. Furthermore, its open-source nature allows for customization, including algorithm validation and autonomous navigation experiments. However, its endurance is limited when carrying substantial payloads, and its performance is affected by environmental conditions, necessitating careful planning. To mitigate these challenges, users can optimize flight paths to minimize power consumption, utilize larger battery packs or alternative power sources, and implement software-based wind compensation techniques to enhance stability in adverse weather conditions. Lastly, although its manufacturing cost ($647.09 USD) is considerably lower than high-end commercial drones, its operation and maintenance demand a certain level of technical expertise, which may pose challenges for beginners.

## CRediT authorship contribution statement

**Franco Alessandro Arenas Mamani:** Investigation, Software, Methodology, Validation, Visualization, Project administration, Formal Analysis, Writing – original draft, Resources. **Gustavo Bryam Capira Malcoaccha:** Conceptualization, Resources, Investigation, Methodology, Software, Formal analysis, Validation, Visualization, Writing – original draft, Writing – review & editing. **Marco Antonio Blanco Quicaño:** Investigation, Methodology, Software, Resources, Formal analysis, Project administration, Writing – Original draft, Writing – review & editing. **Leonardo Gabriel Prado Gutierrez:** Methodology, Investigation, Software, Resources, Formal analysis, Project administration, Writing – original draft, Writing – review & editing. **German Alberto Echaiz Espinoza:** Resources, Methodology, Validation, Supervision, Writing – review & editing. **Erasmo Sulla Espinoza:** Methodology, Resources, Supervision, Writing – review & editing. **Andres Ortiz Salazar:** Methodology, Resources, Supervision, Writing – review & editing.

## Declaration of competing interest

The authors declare that they have no competing financial interests or known personal relationships that could have influenced the work presented in this article.

## Figures and Tables

**Table B.1:** CSV file naming scheme for PID tuning.

PITCH_P_130_S1.csv	PITCH_P_135_I_10_S1.csv	PITCH_P_135_I_20_D_24_S1.csv
PITCH_P_130_S2.csv	PITCH_P_135_I_10_S2.csv	PITCH_P_135_I_20_D_24_S2.csv
PITCH_P_130_S3.csv	PITCH_P_135_I_10_S3.csv	PITCH_P_135_I_20_D_24_S3.csv

**Figure:**
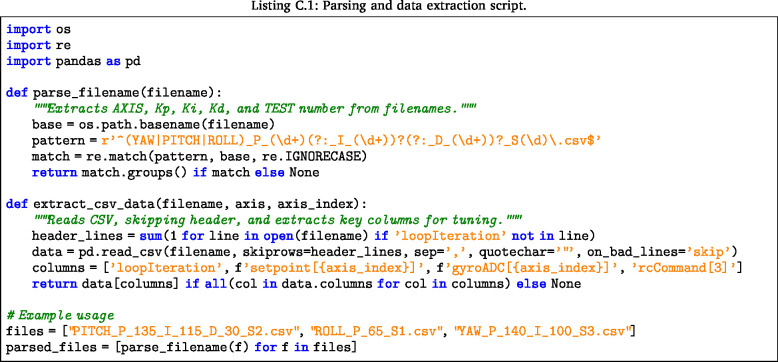


**Figure:**
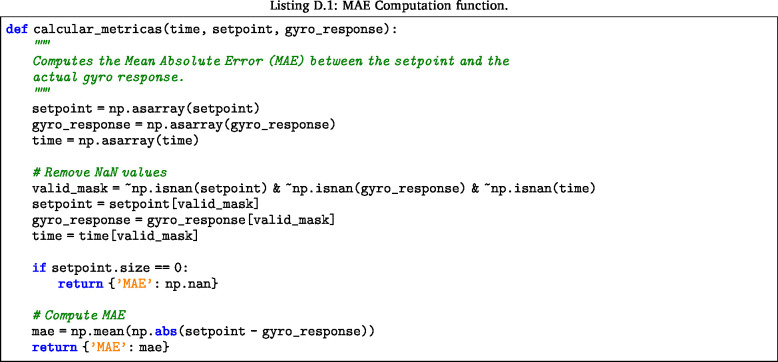


**Figure:**
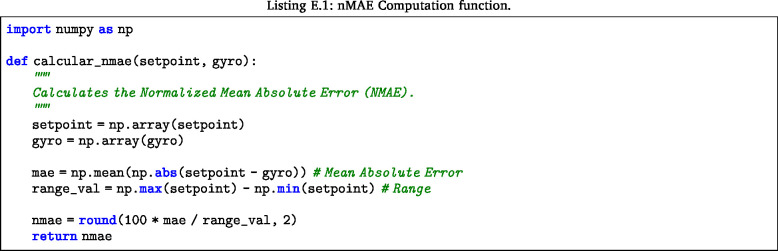


**Figure:**
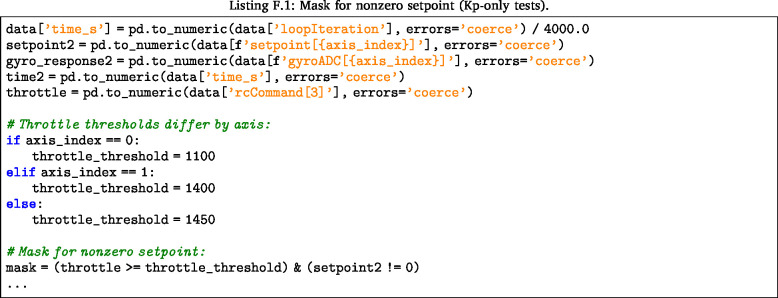


**Fig. F.1:**
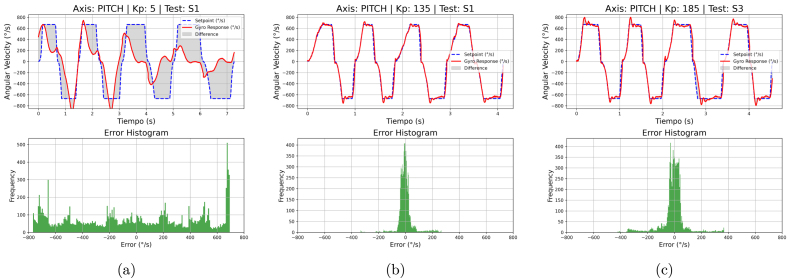
System response for different $K_{p}$ values: (a) insufficient $K_{p}$, MAE = 399.19, (b) appropriate $K_{p}$, MAE = 28.98 and (c) excessive $K_{p}$, MAE = 53.17.

**Figure:**
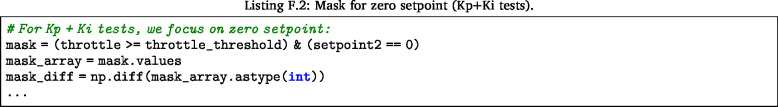


**Fig. F.2:**
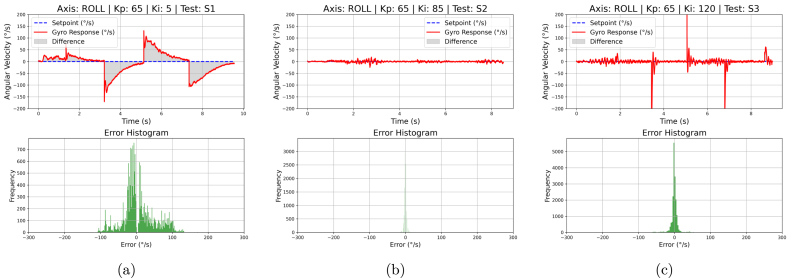
System response for different $K_{i}$ values: (a) insufficient $K_{i}$, MAE = 33.21, (b) appropriate $K_{i}$, MAE = 2.18 and (c) Excessive $K_{i}$, MAE = 6.68.

**Figure:**
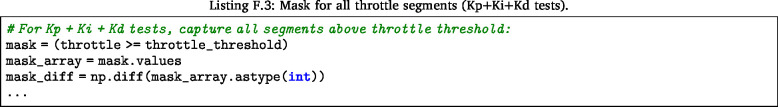


**Table G.1:** Inertia table.

Axis	Test rig inertia ($I_{platform}$)	Drone inertia ($I_{drone}$)	Influence ratio ($I_{platform}/I_{drone}$)
*Roll/Pitch*	$9.56\times 10^{-5}\;{\text{kg m}}^{2}$	$1.41\times 10^{-3}\;{\text{kg m}}^{2}$	6.78%
*Yaw*	$2.29\times 10^{-4}\;{\text{kg m}}^{2}$	$2.48\times 10^{-3}\;{\text{kg m}}^{2}$	9.22%

## Appendix A Supplementary data

In our repository https://doi.org/10.17632/zgsvdtxnfh.2, a demonstration video is available that explains the following aspects:

1. *Drone Assembly and Setup*: Step-by-step guide on assembling the drone, including hardware connections and final setup for flight.
2. *Betaflight Configuration and Blackbox Analysis*: Setting up Betaflight, calibrating the accelerometer, using the Blackbox Explorer, converting BBL files to CSV, and changing motor direction with *BLHeli*.
3. *Charging the FPV LiPo Battery*: Safe charging procedures using a smart charger.
4. *Motor Maintenance*: Disassembling, cleaning, and maintaining the drone motors.
5. *Recording FPV Video from Goggles*: How to capture and record the OSD feed from FPV goggles.
6. *Processing FPV Flight Data with Python*: Implementation of a Python program for analyzing flight data, parameter tuning, and visualization.

## Appendix B CSV file naming scheme for experimental data

During the PID tuning process, flight data was recorded and stored in CSV format. The naming convention for these files follows a structured pattern to facilitate identification and processing. The general format of the filenames is: [AXIS]_[CONTROL TERM]_[VALUE]_[TEST INSTANCE].csv where:

• [AXIS] refers to the control axis being tuned: PITCH, ROLL, or YAW.
• [CONTROL TERM] represents the PID coefficient being adjusted: P (Proportional), I (Integral), or D (Derivative).
• [VALUE] denotes the specific numerical value assigned to the respective control term during the test.
• [TEST INSTANCE] indicates the repetition of the test for statistical validation (S1, S2, or S3).

## Appendix C File name parsing and data extraction

The following Python script automates the parsing of filenames and the extraction of relevant flight data:

1. Extracting PID tuning parameters from filenames.
2. Reading CSV files, skipping unnecessary headers.
3. Filtering relevant data columns to be used for further analysis.

## Appendix D MAE processing and Python code

In this work, the Mean Absolute Error (MAE) is employed to measure PID performance:

(D.1) $$MAE=\frac{1}{n}\overset{n}{\underset{i=1}{\sum }}\left|y_{i}-{\overset{ˆ}{y}}_{i}\right|$$

where n is the number of observations, $y_{i}$ the actual value of the ith observation, and ${\overset{ˆ}{y}}_{i}$ the predicted value of the ith observation. A smaller MAE indicates that the controller closely follows the setpoint, whereas a larger MAE denotes greater deviation.

**Python code example**

## Appendix E Normalized Mean Absolute Error as a percentage (nMAE)

The normalized mean absolute error is calculated using formula (E.1). nMAE is a risk metric based on the expected value of the absolute error.

(E.1) $$nMAE=\frac{1}{n}\overset{n}{\underset{i=1}{\sum }}\frac{1}{c_{norm}}\left|y_{i}-{\overset{ˆ}{y}}_{i}\right|\cdot 100\text{\%}$$

## Appendix F Masking logic for $K_{p}$, $K_{i}$, and $K_{d}$

Below are excerpts showing how masks differ for $K_{p}$ alone, $K_{p}+K_{i}$, and $K_{p}+K_{i}+K_{d}$. These conditions isolate either transient or steady-state segments.

### F.3 Case 3: Proportional–integral–derivative ($K_{p}+K_{i}+K_{d}$)

Final results are completely analyzed on Fig. 22.

## Appendix G Inertia

The complete calculations can be found in our repository https://doi.org/10.17632/zgsvdtxnfh.2.

## Appendix H Glossary

• *PID Controller:* Proportional–integral–derivative controller. PIDs use a closed-loop feedback control mechanism that continuously adjusts outputs based on the difference between a desired setpoint and the measured value.
• *Blackbox:* It is a flight data recorder for FPV (First Person View) drones. The Betaflight Blackbox provides information about flight performance, allowing the drone’s parameters to be adjusted.
• *OSD:* It is the flight information we see in our goggles. It is essential for viewing real-time values that help us understand how the drone is performing at all times.
• *FPV Drone:* The acronym FPV stands for “First Person View”. This means that the meaning of FPV in drones refers to the pilot’s ability to view the scenery from the drone’s perspective during flight, as if they were in the air moving along with the drone.
• *Accelerometer:* Is a device used to measure acceleration in one or more directions. Acceleration refers to the rate of change of an object’s velocity, which can be caused by linear motion or vibrations.
• *Tuning PID:* It is a process that involves finding the values of the proportional (P), integral (I), and derivative (D) parameters of a PID controller to achieve the desired performance.
• *VTX:* It is a video transmitter. It is an essential part of an FPV system. It is a system that sends the video signal from the FPV camera on the drone to an autonomous VRX receiver or one integrated into FPV goggles, so the image captured by the camera can be displayed on a monitor or FPV goggles.
• *Windup:* occurs when the integral term of the PID controller accumulates a significant error over a prolonged period. This typically happens due to control saturation, where the motors reach their power limit and cannot generate additional thrust, yet the controller continues accumulating error. It can also result from excessive corrections, where the drone attempts to adjust its velocity, but the accumulation of the integral term leads to overcompensation, causing oscillations or loss of stability.
• *LiPo* Lithium Polymer (LiPo) battery, a rechargeable battery commonly used in drones due to its high energy density and discharge rates
• *100C*A battery discharge rating that indicates the maximum continuous discharge current relative to the battery’s capacity. A 100C LiPo battery can theoretically deliver a current equal to 100 times its capacity (e.g., a 1300 mAh 100C battery can discharge at 130 A)
